# Not Just a Mother’s Responsibility: Gender, Care and Institutional Influences in Parental Vaccine Hesitancy in Russia

**DOI:** 10.1111/1467-9566.70273

**Published:** 2026-10-01

**Authors:** Ekaterina Borozdina

**Affiliations:** ^1^ Faculty of Social Sciences Tampere University Tampere Finland

## Abstract

The cultural emphasis on mothers’ individual responsibility and choice regarding their children’s health is often considered a key factor behind vaccine hesitancy, especially among the middle class in Western countries. Drawing on qualitative interviews with vaccine‐hesitant Russian mothers, this article broadens this discussion in two ways. First, it shifts the focus from women as autonomous decision‐makers to examining how they share vaccination‐related responsibilities with other family members. Second, it situates these dynamics within Russian healthcare and childcare—institutional settings where neoliberal trends coexist with socialist legacies and strong state control. The study shows that beneath the surface of responsibilised and seemingly individualistic mothering lies a network of care and familial commitment that frames vaccination decision‐making. This is particularly relevant for institutional contexts that promote individual health responsibility without consistently supporting it, thereby fuelling parental distrust in vaccinations and making family involvement essential for navigating volatile welfare environments.

## 1 Introduction

The notion of responsibility is central to sociological research on vaccine hesitancy, as well as to a wider discussion on health in late modernity. Scholars contend that under neoliberal governance responsibility for health is increasingly personalised. It becomes a moral imperative to reflexively manage one’s health risks through informed consumer choices (Crawford 1980). However, this emphasis on individual decision‐making can contradict public health goals: both qualitative and quantitative studies show that “empowered” individuals tend to question institutional constraints, including vaccination requirements, for limiting their autonomy (Hobson‐West 2007; Sanders and Burnett 2019; Cole et al. 2023).

Several recent works have indicated the need for more nuanced and contextualised understandings of the key terms used in vaccine hesitancy research, such as distrust (Nurmi and Jaakola 2023), reflexivity (Numerato et al. 2019) or the hesitancy itself (Schneider‐Kamp 2022; Dubé et al. 2021; Ten Kate et al. 2021). This article focuses on the concept of parental responsibility, using qualitative interviews to examine how Russian vaccine‐hesitant parents interpret and divide responsibility for children’s immunisation. More specifically, I address the perspectives of middle‐class individuals who delay or refuse vaccination out of conscious choice rather than as a result of impeded access to immunisation or lack of knowledge about vaccines.

This study contributes to sociological scholarship on vaccination in two ways. First, it adds to the gender perspective on vaccine hesitancy and parental responsibility. Researchers commonly agree that at the juncture of neoliberalism and gender neotraditionalism, individual responsibility for children’s health falls disproportionately on mothers (Hays 1996). This makes women key decision‐makers on vaccination issues, as well as the central subjects of inquiry into parental vaccine hesitancy (Reich 2014; Dubé et al. 2016; Carrion 2018). The growing number of publications, however, call for broadening the gender perspective on vaccination scepticism by considering the role of other relatives, in particular fathers, in vaccination choices (e.g., McKenzie et al. 2024; Greyson and Bettinger 2022). This research responds to this call by exploring how Russian mothers share responsibilities related to children’s immunisation with their family members.

Second, using the case of vaccine hesitancy, this article engages with a wider sociological debate on how neoliberalism shapes individual responsibility for health. Although sociological research often foregrounds the effects of neoliberalism in healthcare, scholars from different theoretical backgrounds question the overarching character of this framework (e.g., Lupton 1997; Mol 2008; Bell and Green 2016). In regard to responsibility, researchers specifically caution against (1) reducing diverse forms of responsibility to a singular notion tied to individual autonomy and (2) overlooking the contextual specificities of neoliberal responsibilisation across different societies (Trnka and Trundle 2017). In this study, I address both of these points. Rather than depicting mothers as independent choice‐makers, I situate their actions within a broader web of family relations and institutional care arrangements. Moreover, I move beyond established liberal democracies of “the West”—a context where parental vaccine hesitancy has been extensively studied—to a contemporary Russian setting.

Rare studies on vaccination in Central and Eastern Europe indicate that vaccine hesitancy there reflects changes spurred by the dismantling of socialist regimes, most notably changes in state‐citizen relations, gender norms, healthcare institutions and parental agency (Bazylevych 2011; Pop 2016; Hasmanová Marhánková 2017; Janiak et al. 2025). Building on this discussion, I take the case of vaccine hesitancy to explore (gendered) health responsibilisation in contemporary Russia—an environment where neoliberal tendencies intersect with socialist legacies and the authoritarian state’s extensive involvement in health matters (Matveev and Novkunskaya 2022).

### 1.1 Theoretical Framework

Responsibilisation of individuals for their health forms the cornerstone of health governance under late modernity, especially within a neoliberal framework. This process involves several interconnected components. First, health is elevated as a distinct personal value, essential for achieving success in a competitive society and its pursuit becomes a moral duty (Crawford 1980). Second, with the retrenchment of the welfare state, responsibility for health increasingly shifts from institutions to individuals. They are encouraged to act as self‐reliant and “empowered” consumers, who invest effort in traversing the complex healthcare landscape (B. J. Brown and Baker 2012). Finally, advancements in medicine provide the rational foundation for this dynamic: healthcare customers are expected to not just make choices but to strategically manage their health risks, guided by scientific recommendations (Rose 2001).

Health sociologists argue that, in practice, these nudges pull in different directions, because individuals are required both to follow expert advice and to act autonomously. Rather than producing a single model of the neoliberal patient‐consumer, responsibilisation of individuals gives rise to a spectrum of patients’ identities. On one pole of this spectrum are expert patients who actively embrace medicalisation; on the other pole are resisting consumers who assert their agency by challenging biomedical authority (Fox and Ward 2006). The concept of vaccine hesitancy captures this continuum of patient positions with regard to vaccination, specifically focusing on those critical of medical authority.

Vaccine hesitancy is most commonly defined as the delay or refusal of vaccination despite the availability of vaccines (MacDonald and The SAGE Working Group on Vaccine Hesitancy 2015). Social scientists, however, tend to use this term to depart from the behaviourist framing, shifting attention from the dichotomy of vaccine acceptance/rejection to the complex ways in which people make sense of and engage with vaccines (Dubé et al. 2021). They treat vaccine scepticism not as a rigid, irrational belief, but as a plethora of vaccination stances—reflexive, subject to change and producing a range of outcomes, including reluctant immunisation. In this view, vaccine hesitancy refers to a decision‐making process shaped by political, institutional and cultural contexts, rather than to a set attitude (Peretti‐Watel et al. 2015).

Importantly, when it comes to childhood vaccination, this sceptical decision‐making is understood as “a gendered process in which women are responsible for navigating meanings of health, necessity, risk and state intervention for their children” (Reich 2014, 680). From this perspective, vaccination choices—alongside those related to infant feeding or day care—form a site of maternal agency. Mothers demonstrate their commitment to their children’s well‐being by investigating service options, scrutinising expert advice and determining what seems most suitable for their children’s idiosyncratic needs (Reich 2020; Attwell et al. 2018; Ten Kate et al. 2021; Sanders and Burnett 2019).

In this endeavour, various things may spark mothers’ scepticism regarding vaccines. These include, for example, exposure to misinformation on the internet, doubts about pharmaceutical companies’ for‐profit motives, preference for complementary and alternative medicine and parental dissatisfaction with a one‐size‐fits‐all approach to children’s immunisation (Attwell et al. 2018; Numerato et al. 2019; Dubé et al. 2021; Nurmi and Jaakola 2023). Many of these factors can be summarised as a tension between responsibilised mothers and institutions that promote vaccinations. Researchers point to two converging forces behind this tension: the erosion of default trust in state and medical authorities, on the one hand and the neoliberal focus on individual autonomy and consumerism, on the other (Yaqub et al. 2014; Goldenberg 2021).

A growing number of studies, however, problematise such a straightforward understanding of (gendered) parental responsibility, institutions and vaccine hesitancy. Some scholars argue that the labels of “distrust” or “conflict” oversimplify parents’ relationships with institutions. According to these studies, behind parental vaccine criticism there is actually a gradation of institutional distrust (Nurmi and Jaakola 2023). Vaccine‐sceptical mothers often do not categorically reject medicine, but rather strategically navigate it, mixing different sources of knowledge and services as they see fit (Reich 2020). This agency has been particularly well documented among vaccine‐hesitant parents, who use complementary and alternative medicine (Attwell et al. 2018). On the one hand, such individuals doubt allopathic medicine—including vaccines as its products—for being tainted by pharma companies’ interests and creating new health risks. On the other hand, they engage with conventional healthcare if needed, for example, for diagnostics or for treating broken bones.

Other studies highlight social inequalities, in particular class‐ and race‐based ones, that shape both parents’ relationships with institutions and their modes of vaccine scepticism. For privileged mothers, vaccine hesitancy often stems from consumerist empowerment and the ability to make informed choices. In contrast, less advantaged parents may adopt vaccine‐hesitant views as a response to discrimination in welfare access, wanting to protect their children from an unfriendly system (Thornton and Reich 2022; Decoteau and Sweet 2024).

Finally, some publications challenge the view of vaccine hesitancy as an instance of individualistic mothering. Seeing mothers as embedded in their immediate social relations, scholars call for examining the role of wider family in vaccination decisions (Karthigesu et al. 2018; Wachinger et al. 2022; Greyson and Bettinger 2022). Some of these works centre on the notion of care rather than views of individual family members. They trace how, at the micro level, parental vaccine scepticism is connected to childcare practices (McKenzie et al. 2024), and how it is shaped by broader care policy frameworks at the macro level (Hasmanová Marhánková 2025).

In this article, I further elaborate on the complexity of vaccine hesitancy. More specifically, I explore how the contradictory Russian context, which combines neoliberal and statist trends, shapes gendered responsibility in vaccination decision‐making among the middle class.

### 1.2 Healthcare, Parenting and Childhood Vaccination in Russia

The USSR dissolved more than 3 decades ago. However, its legacies—and perhaps more importantly, the reforms that followed its dismantling—continue to shape perspectives on vaccination across the countries exposed to Soviet influence. Studies identify a link between the transition from state socialism to neoliberal capitalism and current high levels of vaccine hesitancy in Eastern and Central Europe (Costa‐Font et al. 2023). These patterns unravel differently in each country. Below, I take a closer look at healthcare and parenting dynamics that form the background for Russians’ vaccination views.

In Russian healthcare, rapid marketisation and shrinking state support have translated into notorious institutional distrust towards medical care, popularity of alternative medicine and proliferation of informal practices (J. V. Brown and Rusinova 2002; Temkina and Rivkin‐Fish 2020; Zvonareva et al. 2018). Authorities attempted to preserve a system of universal health coverage even after the demise of socialist medicine. However, this often came at the cost of service quality (Twigg 2002). Chronic underfunding, outdated equipment and overburdened medical staff have plagued state medical facilities ever since, particularly in areas remote from capital cities. At the same time, the reforms have contributed to the development of commercial medical services, offering the newly emerged middle class a selection of treatment options of higher quality (Cook 2014; Temkina et al. 2022; Denisova et al. 2024).

Sociologists have documented the formation of new—responsibilised and agentic—patients amidst these changes. Some researchers note that wealthy Russians quickly picked up the role of demanding patient‐consumers, already well‐known from “Western” liberal democracies (Temkina 2020). Others argue that neoliberal reforms compelled everybody to adopt a more agentic stance regarding their health. However, the majority exercised agency by finding informal roundabouts within the fragmented health system—rather than by engaging with the healthcare market as “empowered” consumers (Zvonareva and Borozdina 2024).

In the realm of family policies and childcare arrangements, similar tendencies were present until the mid‐2000s. In the 1990s, the Russian state retreated from affluent family support measures to means‐tested policy, making parents feel abandoned by the government (Chernova 2013, 143). As access to state‐funded kindergartens diminished, a “childhood industry” flourished, providing affluent parents with a broad array of commercial childcare and educational options (Shpakovskaya 2015). In this context, Russian mothers increasingly adopted intensive parenting ideals, equating good motherhood with personal responsibility for and investment in their children’s well‐being (Shpakovskaya and Chernova 2022). In matters related to children’s health, such as childbirth and immunisation, this new approach to parenting intertwined with the development of responsibilised patienthood (Temkina and Zdravomyslova 2018; Avdeeva 2020).

Since the mid‐2000s, however, Russian family policy has taken a distinctly pronatalist turn coupled with the promotion of “traditional” gender values on an ideological level. This period is marked by increased state investment in childcare and healthcare, as well as increased control over parental reproductive choices and suppression of a liberal agenda (Rivkin‐Fish 2010; Gradskova 2023). Sociological research suggests that citizens—particularly those in the middle class—respond to this shift with a degree of scepticism. During the decades of ill‐conceived reforms, families have developed distrust towards the government, redefining themselves as autonomous units rather than passive recipients of state paternalistic care (Borozdina et al. 2016; Shpakovskaya and Chernova 2022).

In sum, these institutional and policy tendencies have conditioned the specifics of (middle‐class) parenting in Russia. On the one hand, the intensive mothering ideology and the state’s gender‐conservative ideology paradoxically converge in promoting childcare as women’s prime responsibility. On the other hand, the individualistic orientation of intensive mothering clearly conflicts with the state’s intention to control reproduction. Finally, and above all, whether formulated from a neoliberal or statist perspective, ideological focus on the maternal role is not sufficiently supported on the institutional level. Parents are compelled to cope with this inconsistency on their own—by continuously navigating healthcare and childcare options (Chernova 2012).

This task is often too demanding for mothers to manage alone. As a result, Russian parenting has evolved into a hybrid model, where the sustainability of intensive mothering depends on the support of extended family (Godovannaya and Temkina 2017). Some researchers stress the contradictory nature of such a combination and call for a closer analysis of how parents, in their decision‐making, reconcile the imperatives of intensive mothering with the realities of relatives’ involvement in childcare (Sivak 2018).

Vaccination attitudes and practices build into this environment of parental responsibilisation and institutional distrust. In the Soviet Union, vaccination was treated as a collective duty, not a personal matter and was available to all citizens through state provision (Bazylevych 2011). Russia has largely preserved this pattern. The national vaccination calendar stipulates that all Russian children should receive vaccinations against hepatitis B, tuberculosis, polio, pneumococcal infection, diphtheria, pertussis, tetanus, measles, rubella, mumps and flu (Ministry of Health of the Russian Federation 2014). Additionally, children are expected to undergo an annual Mantoux tuberculin skin test, which is often perceived by parents as a vaccination because the injection is made with a substance of bacterial origin. State clinics administer vaccinations listed in the national calendar free of charge. Parents may also opt for private clinics, which offer a wider range of vaccination services, such as pre‐vaccination allergy testing or the selection of specific vaccines, on a paid basis.

Russian authorities employ institutional means to enforce vaccination. A vaccination certificate is required for enrolment in kindergartens, schools and leisure camps. In cases of infectious disease outbreaks or when children receive the live polio vaccine, unvaccinated children are temporarily excluded from day‐care facilities. However, childhood vaccination is not strictly mandatory—parents can refuse to vaccinate their children.

Statistical data indicate that doubts about vaccines are quite common among the Russian population. Although official sources declare that over 96% of Russian children are fully vaccinated (Rosstat 2023, 68), in a recent all‐Russian survey, only 77% of parents reported fully adhering to the vaccination calendar (WCIOM 2024). In the same poll, when asked about their stance on childhood vaccination, only 43% of respondents chose the option “[I am] for full vaccination”; 53% chose the variant “[I support] only the most necessary vaccinations against selected diseases”.

Qualitative studies have already suggested that, in Russia, scepticism regarding vaccination is rooted in citizens’ distrust of the authoritarian state and the efficiency of its institutions (Kaliszewska and Kołodziejska 2024; Borozdina 2026). This study digs deeper into this issue by examining how, in given institutional circumstances, mothers share vaccination‐related responsibilities with other family members.

## 2 Materials and Methods

### 2.1 Research Sample

This study employs a qualitative methodology to examine vaccine hesitancy in relation to maternal responsibility for children’s health. The research materials consist of 29 semi‐structured interviews with mothers who, in some way, deviated from the national vaccination calendar—either by choosing not to vaccinate their children, delaying vaccination or vaccinating selectively. Participants were recruited through purposive sampling, based on the following criteria.

First, I recruited study participants from the urban middle class—a social group whose decisions about vaccination are deliberate, rather than driven by limited access to vaccines or a lack of knowledge about immunisation. Researchers argue that income level alone may not be sufficient to define social class in Russia (Gontmakher and Ross 2015). Accordingly, I centred on the *professional middle class*, selecting informants with higher education and white‐collar jobs. The resulting sample included individuals working in fields such as architecture, banking, IT, journalism, healthcare and others. Their ages ranged from 29 to 44 years, and they lived either in Moscow or St. Petersburg. Given this sample composition, the study’s findings reflect the views of privileged parents in the country’s major cities. In other segments of the Russian population, vaccine hesitancy may be shaped by different rationales—for example, colonial legacies in Russia’s peripheral republics (Kaliszewska and Kołodziejska 2024).

Second, I aimed to gather data that represents parents’ immediate experiences with Russian childcare and paediatric healthcare services. Therefore, I interviewed mothers who had children below school age or who had just entered primary school—that is, children aged 7 years or younger. In the resulting sample, the number of children per family ranged from one to five.

Finally, I did not use gender as a criterion for selecting informants but asked to speak with the parent responsible for making decisions about vaccination. This recruitment strategy resulted in a sample composed entirely of mothers, 20 of whom were married to or living with a male partner. In addition, I was able to find one man interested in discussing childhood vaccinations. However, he was not simply a middle‐class father of two, but someone involved in vaccination‐related activism. In the interview, we mainly discussed his role in a public campaign opposing the exclusion of unvaccinated children from kindergartens during the measles outbreak.

### 2.2 Interviewing

Interviews were conducted in autumn 2019‐spring 2021. Initially, a call for participants was posted in Russian‐language parental and vaccine‐critical groups on social media. Through this call, people were invited to take part in the research exploring views of vaccine‐sceptical parents. However, because of the sensitivity of the topic, only three individuals responded. Recruitment was further hindered by the COVID‐19 pandemic, as potential participants suspected that the author might expose vaccination critics to the authorities. To ensure trust with informants, I switched to snowball sampling and recruited most participants through my personal social networks.

The interview guide covered key questions about vaccination decision‐making: how parents searched for and evaluated information, how they made choices about different vaccines and how they put those choices into practice. The guide also addressed broader aspects of childcare, such as the division of childcare responsibilities within families and informants’ experiences with Russian healthcare and childcare organisations. I conducted the initial interviews in person but turned to online formats after the onset of the pandemic. In each case, the informant chose the interview location or preferred method of communication. All interviews were recorded, transcribed verbatim and anonymised. The research design was approved by the Ethical Committee of St. Petersburg Sociological Association.

### 2.3 Analysis

I analysed the interviews using ATLAS.ti software and a modified thematic analysis approach (Deterding and Waters 2021). The process began with indexing transcripts according to broad categories informed by the study’s conceptual framework. These included “knowledge about vaccination”, “vaccination attitudes”, “vaccination practices”, “parental responsibility”, “interactions with healthcare institutions” and “interactions with childcare institutions”. At this stage, I also wrote analytical memos to note connections between categories, case‐specific nuances and my preliminary interpretations. After that, I coded selected excerpts using fine‐grained codes derived from the data. This strategy enabled a balance between theoretical grounding and sensitivity to empirical details.

As a result of the analysis, I identified two forms of parental responsibility in vaccination decision‐making: (1) responsibility for navigating vaccination knowledge and (2) responsibility for navigating (healthcare) institutions. Below, I discuss these forms of responsibility, centring on how they are gendered and shaped by the institutional context.

## 3 Findings

### 3.1 Responsibility for Navigating Vaccination Knowledge

Rather than expressing outright critique of biomedicine, the majority of the study participants demonstrated a reflexive stance towards science and its products. They distinguished between the epidemiological approach to vaccination, which emphasises population health risks and their own personalised risk assessments that focus on the well‐being of a particular child. The respondents usually did not oppose vaccination per se and saw healthcare authorities as responsible for using the best medical knowledge to avert epidemics. However, they claimed the right to be discriminatory about vaccination recommendations if following those might cause their own children the slightest harm.

There really are no safe vaccines, and it is really hard to predict immune reactions anyway. If you are the Minister of Health, then you can draw some conclusions based on a sample of several thousand people. But if you are a mother of a particular child… Roughly speaking, I do not want to risk my children at all. Since vaccines have not been fully studied, since their composition has both advantages and disadvantages, I think that as a mother I have the right to choose either to vaccinate my child or not (41 y.o., 4 children)

Several interviewees stressed that they were not “anti‐vaxxers”, because their priority was not contesting vaccination, but catering to their children’s needs through informed decision‐making. They associated parental responsibility with being a filter or adapter between the standardised medical approach and the specifics of children’s (and their families’) circumstances, namely, the nuances of their health condition, housing situation or lifestyle.

You get [medical] help, on the one hand, and on the other hand, you must read everything, filter it, think whether it is necessary and whether it is appropriate. This, in essence, is the responsibility of a parent (34 y.o., 2 children)

According to the interviews, this responsibility for navigating vaccination information mostly rested with mothers. Several respondents justified this allocation by referring to mothers’ “natural” intuition that allows for a deeper understanding of their children. However, most of the study participants evoked functional rather than essentialist reasoning. Because fathers often had higher earnings and focused on breadwinning, it was usually mothers who took parental leave and concentrated on childcare. As a part of this gender‐traditional scenario, women familiarised themselves with a wide range of sources on vaccination—from parental forums on the internet to established biomedical journals—assuming the position of vaccine experts within the family.

My husband, he does not insist on anything. When I get some knowledge [about vaccination], I try to share it with him. However, it is hard for him, he works a lot. When I talk about this topic [childhood vaccination], he is happy to listen, but there are so many nuances. In short, this is my task, I must make up my mind and make a decision (33 y.o., 3 children)

Fathers were neither excluded from the discussion‐making on vaccinations nor exerted direct influence on the final choice. In the interviews, spouses—along with the informants’ parents and in‐laws—were usually depicted as emotionally driven, lacking health‐related knowledge and susceptible to institutional pressure. They often expressed concerns and anxieties in response to mothers’ vaccine‐hesitant stance. This provided women with additional motivation to scan vaccination literature—not only to inform their vaccination choices, but to find the arguments that could soothe their relatives’ fears. Some study participants, who were unable to convince their spouses, reported vaccinating their children out of care for their partners’ feelings. Yet, in most cases, this decision‐making dynamic only reinforced mothers’ expert role.

As for polio, we wanted to go to another kindergarten, but they told us that it would be impossible to get in there without this vaccination (…) Since this topic came up, my husband started fearing polio and tetanus, especially tetanus. [The husband kept asking] “What if he [the child] cuts himself accidently? What if we go to the village, and there [the child gets infected with] tetanus?” So, I had to read about all these (29 y.o., 2 children)

### 3.2 Responsibility for Navigating Institutional Arrangements

In the interviews, mothers’ vaccine hesitancy was tightly intertwined with distrust towards Russian public healthcare and the state in general. The respondents were concerned about the quality and storage conditions of the vaccines, insufficient medical check‐ups before vaccination and physicians’ supposed lack of expertise in the area. The issue of accountability within the health system was specifically problematised. Mothers believed that the authorities pursued vaccination not for public health reasons, but for political motives—mainly, for demonstrating control over the population through the high vaccination rates. Women suspected that state‐employed medical workers were concerned not with patients’ best interests, but with completing nationwide vaccination plans and having paperwork in place. The following quotations illustrate parental distrust in both state‐provided medical services and physicians as the representatives of this system.

Of course, I don’t trust [healthcare] in Russia. I just know that they [healthcare workers] can slip anything anywhere. And it’s scary that they’ll inject this something into your child, and you can’t control it in any way. No matter what, I don’t trust anyone in this clinic (31 y.o., 2 children)

The vast majority of people who get vaccinated, they don’t really think about it—they were told to do it, and they do it. (…) [However, if] something didn’t work out after the vaccination, here [in Russia] we would not be able to prove it. I don’t know of a single case in which the state took responsibility for something that happened [with a child’s health after vaccination] (44 y.o., 1 child)

Mothers’ institutional distrust in healthcare, including vaccination services, prompted them to create tailored health‐seeking trajectories for their children. These solutions were highly heterogeneous. Some respondents opted for a more intensive medicalisation, using private medical services to conduct a thorough child’s health check‐up before selective vaccination. Others tried to establish personalised trust with a particular doctor, finding her via the network of acquaintances. Yet others sought alternative and complementary medicine, like osteopathy and homoeopathy. Notably, all these strategies presumed parental responsibility for navigating Russian healthcare services—a path, the paving of which required knowledge, effort and financial resources.

I believe that we shouldn’t rely on it [public healthcare]. Everyone should do their best to organise health [care] and [medical] consultations for their child (…) If any peculiarities [in child’s health] appear, I have my own plan—who I will go to, when and what I will do (36 y.o., 1 child)

Such navigation of the Russian healthcare landscape was, however, a rather difficult task for a mother to accomplish independently. In their interactions with the healthcare system, the interviewees often relied on financial support from their partners, who covered expenditures on paid medical services, including vaccinations. Moreover, fathers took on part of the responsibility for institutional interactions, in particular, those interactions that required extra assertiveness from the parent. For instance, fathers were ensuring access to preferred healthcare services, negotiating with insurance companies to cover additional pre‐vaccination tests, handling vaccination refusal documentation and similar matters. Activism demonstrated by the only interviewed father fits into this pattern of outward‐oriented agency quite well. Below, an excerpt from an interview with a mother shows how her ex‐husband’s involvement—his financial contribution and efforts in selecting healthcare providers—enabled a personalised vaccination scenario for their child.

A: He [the respondent’s ex‐husband] makes all the decisions regarding [the child’s] education and vacations. I mean, he sponsors these because I work in a charity and can’t afford all these.

Q: And what about issues related to the child’s health, like medical treatment or vaccination?

A: We decide on all these together. About vaccinations, we took some of the consultations at [name of the private clinic]. (…) Then we checked this with other doctors through this special [online] service, with doctors who do not even live in this country. (…) Then I gave him [the ex‐husband] a task to find the clinic where we would do the vaccination so that they stored and transported vaccines properly. In tasks like these, he can make the dead move (36 y.o., 1 child)

Because Russian public kindergartens—the most accessible childcare option—require vaccination, vaccine‐hesitant parents had to develop complex strategies not only for healthcare but also for day care. Several mothers reported leaving their jobs or reducing their working hours to stay with children at home. Others turned to private kindergartens, which tolerate unvaccinated children or made informal arrangements with staff of public kindergartens. Grandmothers’ involvement through babysitting was another resource mobilised by the interviewees. The following quotations illustrate the considerable efforts mothers invested in building and maintaining this network of childcare solutions, while also underscoring its precarity. Any change in family circumstances—be it reduced income, altered work schedules or declining health of grandparents—could force informants to turn to public kindergarten services, thereby prompting at least partial vaccination of their children.

He [the respondent’s son] goes to the state kindergarten, but he goes there ostensibly, so to speak [the child is formally registered in the kindergarten but does not actually attend it]. We probably don’t even need kindergarten. We have three grandmothers; we have someone to look after the child. But the thing is that the grandmothers have their health [issues]. And there is my work. Now I have such a work schedule that we can plan who [of the family members] looks after the child. I’m at home twice a week. But it [the work schedule] is hard to predict, and I can’t give up kindergarten completely (44 y.o., 1 child)

We did the first vaccination when we went to the kindergarten, to the state one. We attended another [private] kindergarten without vaccinations, and basically no one required it there. When we had to go to a state kindergarten [due to the family’s worsened financial situation]… Well, by that time I myself already thought that probably we should start doing something [getting the child vaccinated] (29 y.o., 2 children)

These excerpts also emphasise that relatives, although not central to making vaccination choices, played an important role in implementing those decisions. The sustainability of complex healthcare and childcare strategies depended on, for instance, fathers covering private medical services or grandparents participating in babysitting.

## 4 Discussion

This study contributes to the prolific scholarship on gendered responsibility in vaccination decision‐making. Literature on the subject typically connects vaccine hesitancy to the tension between personal health choices and public health mandates in liberal democracies of “the West”, depicting mothers as the primary agents of responsibility in the case of childhood vaccinations (Reich 2014). This research expands the scope of analysis in two ways. First, it shifts the focus from individual women to the broader familial dynamics of responsibility‐sharing. Second, it contextualises both vaccine hesitancy and parental responsibility within the rarely considered Russian setting, where neoliberal responsibilisation coexists with authoritarian rule and incoherent post‐socialist welfare.

The findings confirm the well‐documented pattern that, under neoliberal governance, parental vaccine hesitancy often emerges as an unintended consequence of individual health responsibilisation (Reich 2014; Sanders and Burnett 2019; Cole et al. 2023). The mothers I spoke with tried to avoid standardised approaches to managing their children’s health—be it medical recommendations for vaccination or conventional pathways within the healthcare system. The study, however, adds nuance to the picture by foregrounding the role of the institutions in shaping specific modes of health responsibilisation and vaccine scepticism. Russian healthcare, formed by continuous and incoherent reforms, is known for fostering institutional distrust among its inhabitants (J. V. Brown and Rusinova 2002; Cook 2014; Zvonareva and Borozdina 2024). Although this distrust readily translates into parental vaccine hesitancy, the pattern of this translation differs from what has been described regarding Western Europe and the US. Most notably, my interviewees felt compelled—rather than empowered—to take responsibility for their children’s health, including vaccination.

In practice, taking on such responsibility took different forms. Some parents, seeking a wider selection of care options, turned to private healthcare. Others, preferring “natural” approaches, opted for alternative or complementary treatments. Yet, despite their differences, these choices stemmed from a common root—the distrust of Russian public healthcare, viewed both as a vehicle of state control and as falling short of middle‐class service expectations. As a result, the pattern of somewhat compelled parental agency has developed. This pattern reflects an institutional environment that inconsistently blends neoliberalism and statism, encouraging individual health responsibility but failing to steadily support it.

The article also complicates the gender perspective on vaccination responsibility and vaccine hesitancy. Similar to earlier research (Reich 2014), this study shows that women, in line with traditional gender roles, assume primary responsibility for decisions regarding children’s well‐being. However, fathers and other family members are not absent from the decision‐making process. Although mothers take the lead in exploring information on vaccines and making vaccination choices, relatives’ concerns and pro‐vaccination views prevent vaccine refusal from becoming a settled position.

Contrary to some of the previous findings (Wachinger et al. 2022), relatives’ influence in my sample was not straightforward. Family members’ involvement did not directly challenge mothers’ vaccination decisions but encouraged women to further explore the literature. Occasionally, respondents chose to vaccinate their child to ease the anxieties of husbands or grandparents. However, a pattern that appeared more consistently was the reinforcement of mothers’ role as principal experts on children’s wellbeing and emotional caretakers (not only for children, but for all family members).

Traditional gender expectations also conditioned relatives’ participation in vaccination decision‐making, particularly in carrying out the decisions. Previous research has shown that vaccine hesitancy demands additional effort from parents when engaging with healthcare, day‐care and educational systems. For example, individuals who refuse vaccines must seek legal exemptions (Reich 2018), and those opting for selective vaccination invest extra time and resources in developing tailored vaccination plans for their children (Ten Kate et al. 2021). In the Russian welfare environment, macro‐level contradictions between statist and neoliberal logics, as well as inconsistent quality of services, complicate things for all families (Chernova 2012; Isupova 2024). For vaccine‐hesitant parents, these challenges become even more acute.

My interview materials show that even those vaccine‐hesitant mothers who belonged to the middle class faced difficulties in navigating healthcare and day‐care options independently. Consequently, women partially shared this burden with their spouses. The interviewees positively assessed their partners’ participation in vaccination matters, perceiving it as a sign of more involved fatherhood. However, the allocation of responsibilities within families still adhered to the traditional division of gender roles, with husbands taking on tasks associated with financial provision and protecting the family’s rights in public. Grandmothers’ involvement in babysitting offered another example of how relatives contributed in gender‐traditional ways to the feasibility of vaccine‐hesitant parenting.

It is crucial to remember, though, that this study’s findings are based on interviews with mothers and thus reflect their perspective. In my interviewees’ accounts, men appear mostly as supporting figures, but further analysis of how fathers themselves see their roles is needed. It is likely that their views on childhood vaccination and father’s responsibility are more complex and diverse than portrayed by mothers. The only interview with a father, who was also an activist, suggests that men can develop an informed and agentic stance on vaccination. A similar pattern—that is, that mostly mothers make vaccination decisions, but some fathers actively participate in decision‐making work and care work—has been noted by previous research (McKenzie et al. 2024). Yet, the case of committed vaccine‐hesitant fathers still awaits further exploration.

Moreover, there is no single “maternal perspective”. There is a variation in mothers’ vaccination views shaped by class‐, race‐ and ethnicity‐based inequalities, as well as by regional diversity (Thornton and Reich 2022; Decoteau and Sweet 2024; Kaliszewska and Kołodziejska 2024). This study by design focuses on relatively privileged mothers and therefore does not attend to those differences. Its findings are informative only about vaccination decision‐making and responsibility dynamics among the urban middle class.

Finally, the research brings to light how mothers’ individual responsibility for children’s health is, in fact, tightly entangled with care relations and arrangements. Sociological publications on vaccine hesitancy have recently delineated the link between vaccination and care, arguing that parental decision‐making should be considered within the context of routine care work, as well as wider care policies (McKenzie et al. 2024; Hasmanová Marhánková 2025). This article adds to this discussion. It shows that Russian mothers, although adopting the ideology of intensive parenting, also carry a broader spectrum of responsibilities for the well‐being of their relatives. It also demonstrates that parental vaccination choices tightly intertwine with their childcare choices. In sum, these indicate that beneath the surface of responsibilised and seemingly individualistic mothering lay a network of care and commitment that framed both mothers’ vaccination decision‐making and the ways in which those decisions were carried out. In a more theoretical sense, this finding suggests that gender‐sensitive analysis of vaccine hesitancy may benefit from moving beyond a rigid binary of ‘female’ and ‘male’ responsibilities and stances. Instead of viewing family members as isolated actors, it might be valuable to consider the relational dynamics of care that underpin vaccination choices.

## Author Contributions

**Ekaterina Borozdina:** conceptualisation, writing – original draft, writing – review and editing, funding acquisition, formal analysis, methodology.

## Funding

The data collection was funded by the Russian Science Foundation (Project No 19‐78‐10128).

## Conflicts of Interest

The author declares no conflicts of interest.

## Data Availability

Research data are not shared.
